# The GP Evidence website: focus-group evaluation study of a new point-of-care information resource

**DOI:** 10.3399/BJGPO.2025.0109

**Published:** 2026-06-17

**Authors:** Julian Stephen Treadwell, Joanna C. Crocker, Kamal R. Mahtani

**Affiliations:** 1 Nuffield Department of Primary Care Health Sciences, University of Oxford, Oxford, UK; 2 Centre for Academic Primary Care, University of Bristol, Bristol, UK; 3 National Institute for Health and Care Research (NIHR) Oxford Biomedical Research Centre, Oxford, UK

**Keywords:** clinical decision-making, evidence-based practice, primary health care

## Abstract

**Background:**

The GP Evidence website is a new point-of-care information resource aimed at GPs, providing quantitative information on the benefits and harms of treatments for common long-term conditions (LTCs), an identified gap in clinicians’ knowledge.

**Aim:**

To explore whether the clinical content in GP Evidence can be accessed and understood by GPs in a way that can be integrated into their clinical thinking and decision making.

**Design & setting:**

Online questionnaire and focus group with British GPs.

**Method:**

Online focus groups were undertaken. Within these, participants were first invited to consider hypothetical treatment decisions based on fictional clinical vignettes, they were then asked to give individual responses about their anticipated consultation and treatment intentions in an online survey. This was followed by an online semi-structured focus group discussion. Qualitative data were analysed thematically, and quantitative data presented as summary statistics.

**Results:**

In the main, the clinical content of GP Evidence was easily understood by GPs. Changes in intended prescribing and discussions with patients were described, as well as increases in confidence. Some barriers to use were identified mainly relating to competing system pressures.

**Conclusion:**

This study confirms the usability of GP Evidence and suggests it has potential to introduce new information and influence practice. This could support shared decision making and person-centred practice. It is a preliminary study with limitations, and further research is needed to assess its impact in real-world settings.

## How this fits in

GPs report difficulty understanding treatment effect sizes, and clinical guidelines rarely present this information in a user-friendly way. The website GP Evidence was designed to present these data clearly at the point of care. This early evaluation study suggests the resource is usable, can improve clinician understanding and confidence, with potential to inform discussions with patients and prescribing decisions. Its integration into practice could enhance the use of clinical guidelines and facilitate shared decision making.

## Introduction

The GP Evidence website (www.gpevidence.org) is a new point-of-care information resource aimed at GPs and other healthcare professionals. Its principal aim is to provide easily understandable quantitative information on the benefits and harms of treatments for common long-term conditions (LTCs) to support shared decision making (SDM). This means information such as the absolute risk reduction in cardiovascular events conferred by a statin, or the number-needed-to-treat with spironolactone to prevent death in heart failure. International research shows that specialists and generalists have a poor understanding of such information;^1^ this has also been shown among British GPs.^2^ This information is prohibitively difficult for busy clinicians to access and is not included in clinical guidelines.^3,4^ Our research with British GPs^5^ showed an awareness of this knowledge deficit and recognition that it limits ability to provide person-centred care: without an understanding of the likelihood of benefits or harms of treatments, it is difficult to answer questions such as ‘What are the pros and cons of this drug for me doctor?’. Similarly, prioritising options in complex polypharmacy is impossible.^3,6,7^

Although this quantitative information is theoretically straightforward, there are recognised barriers to its use: clinicians’ low confidence in statistical and scientific terminology, time constraints, competing system pressures such as clinical guidelines, performance metrics, and medico-legal fear.^5,8–13^ To overcome these, GP Evidence was developed using participatory co-design methods with GPs and patients.^14^ The resulting design uses infographics, extremely plain language, layered text, and information architecture aligning with GPs’ mental maps and the broad structure of guidelines from the National Institute for Health and Care Excellence (NICE). Scientific content is derived from high-quality evidence syntheses, principally NICE and Cochrane systematic reviews.^15^

The website is designed for rapid, in-consultation use but also contains short-read textual content. Launched in February 2023, it contains information on 45 treatments for 12 common LTCs and, as of September 2025, has received more than 72 000 unique visitors from 74 countries. NICE link to it as a decision-support resource from two recently updated guidelines.^16,17^

User-testing during development generated feedback from GPs which informed the iterative design process. Finally, we conducted a preliminary evaluation study on the completed website with another group of GPs who were naïve to the product which we report here.

### Patient and public involvement

Patient and public input occurred for the duration of the GP Evidence project between 2017 and 2023. At the outset, two focus groups involving 14 adults living with LTCs advised on project aims, priorities, and value. A one-day workshop with three patient and public involvement (PPI) members and four evidence synthesis experts was held early in the website design process to co-design a strategy for the collation of clinical evidence and presentation of content for the website. This group went on to form a steering committee for the duration of the project, advising on dilemmas and options regarding the presentation of evidence as well as being involved in discussions about this evaluation study.

### Study aim

To explore whether the clinical content in GP Evidenc*e* can be accessed and understood by GPs in a way that can be integrated into their clinical thinking and decision making.

## Method

### Online focus groups with embedded online survey

Four 90-minute online focus groups were conducted with 15 GPs in total between November 2022 and January 2023. For the first half of the sessions, GPs were asked to work individually on an online survey employing fictional clinical vignettes to explore their prescribing intentions. They were then given access to GP Evidence and asked to repeat the questions. The group was then brought together for a semi-structured discussion.

### Recruitment

Sample size was influenced by feasibility and resources available for this preliminary study as part of a wider project. We did not aim to acquire generalisable quantitative data nor attempt purposive or maximum variation sampling.

Participants were recruited from a pool of 213 GPs who had completed an online survey in an earlier phase of the research.^2^ Originally recruited via widely distributed email invitations, they had demographic characteristics broadly representative of the UK GP population (Supplementary Table 1). On invitation they received a participant information document and consent form that was completed before participation. They were offered a £75 gift voucher as an incentive. Inclusion and exclusion criteria are described in Table 1.

**Table 1.** Inclusion and exclusion criteria

Inclusion criterion: GPs currently practising in the UK NHS Exclusion criteria: GPs who have not been in NHS clinical practice within the past year GPs still completing specialist training (ST1–5) GPs with further training, experience, or roles that may confer expertise about the subject matter GPs who had any previous involvement with the design of GP Evidence

### Design of study and materials

The online questionnaire (Supplementary Table 2) was designed to assess the comprehensibility of information from GP Evidence and its possible effect on prescribing behaviour in three differing clinical situations. First, the option of statins and blood pressure-lowering drugs in the primary prevention of cardiovascular disease, where we anticipated the quantitative benefits of treatments would be variably known by GPs and the effect of new knowledge on prescribing unpredictable. Second, the role of angiotensin-converting enzyme (ACE) inhibitors in chronic kidney disease where we were aware of an existing knowledge deficit^2^ and where new knowledge would be highly likely to change prescribing. Finally, a multiple long-term conditions (MLTC) or polypharmacy case with several treatment options, a range of information to explore on GP Evidence and anticipated high variability in intended prescribing.

Participants were asked to state their prescribing intention on a three-point Likert scale, confidence in that decision on a five-point Likert scale and invited to make free-text comments. Group discussion was conducted using a guide shown in Supplementary Box 1.

A pilot study was conducted face to face with a convenience sample group of 11 GPs not previously involved in the research. None of these participated in the main study. Adjustments were made to the questionnaire and structure of the discussion session.

### Data handling

Data handling was conducted according to University of Oxford policy.^18^ Research data were collected without identifying data. Focus groups were conducted over Microsoft Teams. The questionnaire was conducted using Jisc Online Surveys with responses downloaded into Microsoft Excel. Audio-recordings were captured using the transcription function of Microsoft Teams. Transcripts were checked for errors, amended then saved on Microsoft Word documents. Qualitative data were analysed within NVivo (version 12) software.

### Analysis

#### Qualitative data

Descriptive thematic analysis was undertaken.^19^ Data from transcripts and survey were analysed together. Coding was inductive. Final codes with definitions are in Supplementary Table 3. Categorisation of codes into themes was achieved using the ‘one-sheet-of-paper’ technique.^20^ Analysis was undertaken by one researcher (JT) who applied reflexivity during the analytic process,^21^ paying attention to their own assumptions and perspective. Regard was given to their (non-pecuniary) vested interest in the project, and the strong likelihood of participants’ social instinct to give positive feedback to a researcher they knew had been involved in the design of the website.

#### Quantitative data

Quantitative responses from the survey were collated in Microsoft Word and summary tables created.

## Results

Seventeen GPs responded to the invitation and 15 were able to join a focus group. A low response rate precluded selective maximum variation sampling. Focus groups were organised by participant availability. Participant characteristics are detailed in Table 2.

**Table 2.** Characteristics of GP participants

Characteristic *n* = 15	
**Sex**	
Female	10
Male	5
**Age, years**	
30–39	2
40–49	2
50–59	7
≥60	4
**GP role**	
GP principal	3
Salaried GP	3
Locum GP	9
**Place of original medical degree**	
UK	14
Non-UK	1
**Geographical region**	
North-West England	2
Greater London	3
South-East England	3
South-West England	6
Scotland	1
**GP description of practice**	
Urban	6
Rural	2
Mixed urban–rural	7

### Qualitative findings

Findings were grouped into four themes (positive reactions and effects; patient interaction; complexity; and barriers and negative perceptions) with sub-themes (in bold below); key quotations are shown in Table 3. Further quotations are shown in Supplementary Table 4.

#### Positive reactions and effects

##### Positive reactions

All participants expressed positivity about the value of GP Evidence to support practice.

##### New knowledge or change in prescribing intention

Participants described acquiring new knowledge, which appeared to have been understood within the context of their existing knowledge and perspective. Many described shifts in their prescribing intentions or approach to conversations with patients.

##### Confidence

Some participants gave examples of new information increasing their confidence, even though their initial decision remained unchanged.

Mostly, information acquisition appeared to have been easy, supported by clarity of website design. There were no comments about time constraints when gathering single-treatment-related information.

### Patient interaction

Descriptions were offered regarding how GP Evidence might support conversations with patients, either by sharing infographics from the website directly in consultation or using new components of knowledge to inform the consultation.

### Complexity

#### Multiple long-term conditions (MLTCs) and polypharmacy

Addressing the complexity of MLTCs and polypharmacy was described as more challenging than single-treatment decisions. Some regarded this as almost too challenging; quotes in Table 3 illustrate differing reactions, one GP describes using gut feeling to support decisions, another speculates on how a potentially more advanced version of GP Evidence might help, another describes how GP Evidence might help in its current form. In the online survey, for the case on MLTCs or polypharmacy, most answers indicated specific learnings about individual treatments.

**Table 3.** Themes and quotations from online survey and focus groups

**Theme 1: Positive reactions and effects** *‘Yeah, this is completely game-changing it is so good … and I'm not trying to be smarmy. For years I've loved the concepts of shared decision making and helping people with their goals and wanting to have these discussions.’* (Participant 4) *‘It’s fantastic to have a place where all the evidence is gathered together and I'm not having to be the person who reads all the latest guidance.’* (Participant 7) *‘I think before the website I was more inclined to give her a blood pressure medication, less inclined to give her a statin. And having looked at the website, I was thinking probably either the other way around or give her both.’* (Participant 6) *‘I think for me the main thing was that it made me more confident in my decisions ... and perhaps made me more coherent in my reasoning.’* (Participant 10)
**Theme 2: Patient interaction** ‘*The statin chart helped me realise the benefit and would help in consult to explain to patients.’* (Participant 13, text response) *‘… now I'm clicking on the button, and I think that would be so useful to share with patients, you know, the impact of aerobic exercise. And I'd quite like to compare that with the impact of medication, for example …’* (Participant 8)
**Theme 3: Complexity** *‘… it it’s that really complex group where it’s much harder to try and draw together, if we've got drugs which are treating two or three things. So if you've got cardiovascular disease and you're on bisoprolol and you've got some benefit for your heart, some benefit for your AF speed, some benefit for your heart failure, some benefit for blood pressure, but smaller … those are always the most complex bits of work … and then it’s still almost impossible to get the data that you need to do anything other than a … gut feeling.’* (Participant 5) ‘*…for example, that patient was on a big dose of metformin, so potentially renal effects, if the DAP* [dapagliflozin] *would give some benefit on the diabetes. So that maybe that* [the metformin] *could be reduced a little bit and they'd need less of their beta blocker in total. Can we end up with less tablets …and that’s a kind of more convoluted pharmaceutical algorithm probably which … we're not there yet, but it’s the kind of thing which you can imagine the development on a program like this would be able to say …’* (Participant 7) ‘*… playing with the site would give better familiarity with the links and the ways to sort of jump between the different sections. So, I think that that is something that with longer to play with it, we'd get better at … utilizing that functionality.’* (Participant 5)
**Theme 4: Barriers and negative perceptions** *‘I think the thing that will probably confuse or worry me would be the bit where it says say for this type of evidence “quality low to very low …” What does that actually mean in in practice? I'm sort of struggling … is that just an extra level of complication in this maybe?’* (Participant 14) *‘… it looks like a good tool, but one would have to be using it a lot before it became a good tool, I'd say. And just using it straight off … it just engendered … like sense of panic and of uh, that makes my knowledge even worse.’* (Participant 15) *‘The blood pressure benefits/harms on website would clearly suggest not much benefit to treating but we are hampered by QoF/ NICE guidelines pushing into treatment, and cardiology! Was quite surprised. Creates an ethical dilemma re recommending.’* (Participant 11, text response)

#### Further exploration of website

Some participants suggested that with increasing familiarity and use of GP Evidence, navigating this complexity may become easier over time.

#### Accumulating knowledge

There were speculations that an accumulation of knowledge may occur over time, avoiding having to look up individual pieces of information in complex situations in the future.

### Barriers and negative perceptions

#### Confusion

Confusion arose for some about particular aspects of content. One example was the implication of ‘low-quality’ evidence rating and how that should affect practice. One GP found the volume of information was too great, engendering a sense of panic.

#### Challenge to perceptions of normative practice

Challenges were described regarding the implementation of any changes in behaviour arising from new knowledge. A common issue was how information from GP Evidence fits with NICE guidelines or the Quality and Outcomes Framework.

#### Barriers related to clinical autonomy

Barriers associated with clinical autonomy were reported; for example, the role of other healthcare professionals in managing LTCs in primary care.

### Quantitative findings

Table 4 shows results from the questionnaire on intention to prescribe and confidence levels before and after using GP Evidence. Eighty-four quantitative answers were provided in total by the 15 GPs (six answers were lost owing to user-error). Thirty-eight of these 84 revealed some degree of change in intention to prescribe. For questions about confidence, one-quarter of answers indicated an increase in confidence; only one reported a decrease.

**Table 4.** Summary of participants’ responses to intention to prescribe and confidence questions on online survey

	Decrease		No change	Increase	
**Number of points change on Likert scale**	**-2**	**-1**	**0**	**+1**	**+2**
Q1a) Likelihood of prescribing drug treatment for hypertension	4	3	7	0	0
Q1b) Likelihood of prescribing statin	1	0	10	2	1
Q1c) Confidence	1	0	8	5	0
Q2a) Likelihood of aiming for lower blood pressure target	4	1	9	0	0
Q2b) Likelihood of prescribing ACE inhibitor	5	2	7	0	0
Q1c) Confidence	0	0	5	6	3

Full description of questions and Likert scales in Supplementary Table 2. Figure in each cell describes the number of participants with a particular number of steps of change on the relevant Likert scale in their answers before and after exposure to GP Evidence.

*n *= 14 (one participant’s quantitative answers were unavailable owing to an error in data entry). ACE = angiotensin-converting enzyme

## Discussion

### Summary

All participants responded positively to GP Evidence, perceiving it as a usable tool able to provide them with useful, understandable information that could support their practice. They described several hypothetical changes to their prescribing intentions and reported increases in confidence in decisions. There were also some expressions of uncertainty about some content within GP Evidence. The application of new information to a theoretical example of MLTCs or polypharmacy appeared to be more challenging.

### Strengths and limitations

Strengths of the study are that the participant group contained GPs with a wide range of demographic and professional characteristics, although there were proportionally more female GPs and GP locums than the general GP population. Although the sample size was small, at this level of analysis, qualitative data saturation seemed to have been reached, with no new codes added after coding the third focus group transcript. Study design enabled the capture of individual GPs’ responses unaffected by others, as well as wider ranging responses facilitated by focus-group discussion. The questionnaire assessed responses to both simple and complex clinical scenarios.

Limitations of the study include a probable response bias driven by self-selection of participants likely to be interested or favourably disposed to GP Evidence. Participants would have been likely to give positive responses to an interviewer who they knew was involved in the website’s development. We aimed to reduce this by clearly inviting criticism and applying researcher reflexivity.

Although the vignette-based questionnaire was designed to reflect everyday clinical work, the artificial nature of answering theoretical questions within the protected context of the study limits the assumptions that can be made about what might happen in practice. We could not study any effects on long-term knowledge retention, behaviour change, or clinical outcomes.

The data size and analysis were limited as this was a small-scale preliminary study. We did not include triangulation methods or independent coding of qualitative data owing to resource limitations. We did not attempt a deeper analysis of issues such as decision making in complexity, the interaction of new knowledge with systemic drivers and barriers, or questions of power and professional freedom. Quantitative data are only able to describe the outcome for this study and are not generalisable to a wider GP population within or outside the UK.

### Comparison with existing literature

Research efforts to address the long-standing challenge of improving the quality of shared decision making in practice have focused mainly on the development of patient-facing decision aids, consultation skills, and models to support their use.^22–24^ This is a well-developed field and although decision-aids have been shown to improve patients’ knowledge, risk perceptions, and ability to engage in decision making,^25^ their effective use remains difficult to implement.^26–28^ Clinician-facing resources, such as GP Evidence, are less common than patient-facing resources although other examples do exist.^29,30^ Clinicians and GPs do make effective use of online information resources^31–33^ and a recent study highlighted the need among GPs for tools to help with LTC management. Evidence-based practice teaching has traditionally focused on scientifically oriented aspects such as study design and critical appraisal.^34^ However, in recent years there is an acknowledgement that for practising clinicians, a focus on understanding treatment effect sizes and applicability of evidence to individuals would be of more value than traditional critical appraisal.^35^ Tools such as GP Evidence support such a model of education and practice and offer a new model to improve shared decision making.

### Implications for research and practice

GP Evidence offers summary information on the benefits and harms of treatments for LTCs, which has hitherto been difficult for clinicians to access and understand. Within the limitations described, this study supports the idea that GPs can understand and assimilate this with relative ease and apply the new knowledge to clinical practice. This was most apparent for single-condition, single-treatment scenarios. In the context of MLTCs and polypharmacy, this information seems more challenging to integrate, although participants in this study suggested that this may occur over the longer term with further exposure to website content. This new understanding has the potential to support better shared decision making between clinicians and patients.

The design of GP Evidence enabled participants to understand population-level results from clinical research, overcoming barriers of statistical illiteracy or poor confidence in interpreting scientific literature. This suggests that it may have potential as a teaching tool to educate about the interpretation of scientific literature and its application in practice. Further educational research could explore this possibility.

This study only tested theoretical questions; further research is needed to understand how GP Evidence affects practice in the real world. In clinical practice, numerous barriers exist to the use of new scientific information: whether information will be accessed at all, whether clinicians feel interested or able to change their decision making in the light of multiple competing interests, whether they feel able to communicate this with patients, and how patients may respond to any new offers of choice or shared decision making. We plan a realist evaluation study to answer these questions. This will also provide information regarding the feasibility of conducting a quantitative study to measure the effects of GP Evidence use on prescribing rates or clinical outcomes.

## Data

Research data supporting this study cannot be made available as participants did not consent for their data to be shared. However, the corresponding author welcomes any enquiries about the study findings and will respond to reasonable requests within the limits of ethical/consent restrictions.

## References

[1] Hoffmann TC, Del Mar C (2017). Clinicians’ expectations of the benefits and harms of treatments, screening, and tests: a systematic review. JAMA Intern Med.

[2] Treadwell JS, Wong G, Milburn-Curtis C (2020). GPs’ understanding of the benefits and harms of treatments for long-term conditions: an online survey. BJGP Open.

[3] Guthrie B, Payne K, Alderson P (2012). Adapting clinical guidelines to take account of multimorbidity. BMJ.

[4] McCormack JP, Martin SA, Newman DH (2015). Comment on Inzucchi et al. Management of hyperglycemia in type 2 diabetes, 2015: a patient-centered approach. Update to a Position Statement of the American Diabetes Association and the European Association for the Study of Diabetes. Diabetes Care 2015;38:140–149. Diabetes Care.

[5] Treadwell J, Crocker J, Rushforth A (2021). GPs’ use and understanding of the benefits and harms of treatments for long-term conditions: a qualitative interview study. Br J Gen Pract.

[6] Treadwell J (2015). Coping with complexity: working beyond the guidelines for patients with multimorbidities. J Comorb.

[7] Duerden M, Avery T, Payne R (2013). Polypharmacy and medicines optimisation: making it safe and sound.

[8] Wegwarth O, Gigerenzer G, Gigerenzer G, Muir Gray JA (2011). Better doctors, better patients, better decisions: envisioning health care 2020.

[9] Carlsen B, Norheim OF (2008). “What lies beneath it all?”—an interview study of GPs’ attitudes to the use of guidelines. BMC Health Serv Res.

[10] Solomon J, Raynor DK, Knapp P, Atkin K (2012). The compatibility of prescribing guidelines and the doctor–patient partnership: a primary care mixed-methods study. Br J Gen Pract.

[11] Elwyn G, Wieringa S, Greenhalgh T (2016). Clinical encounters in the post-guidelines era. BMJ.

[12] Neumann I, Alonso-Coello P, Vandvik PO (2018). Do clinicians want recommendations? A multicenter study comparing evidence summaries with and without GRADE recommendations. J Clin Epidemiol.

[13] Brown EL, Poltawski L, Pitchforth E (2022). Shared decision making between older people with multimorbidity and GPs: a qualitative study. Br J Gen Pract.

[14] Treadwell J (2023). Improving GPs’ knowledge of the benefits and harms of treatment to support decision making in multimorbidity: qualitative research and co-design of a novel electronic information resource [doctoral thesis].

[15] Treadwell J, Armitage LC, Collins KK (2025). Co-production and implementation of an evidence collation strategy for a novel point-of-care information resource: gpevidence.org. BMJ Evid Based Med.

[16] National Institiute for Health and Care Excellence (NICE) (2024). Menopause: identification and management [NICE guideline NG23]. https://www.nice.org.uk/guidance/ng23.

[17] NICE (2023). Cardiovascular disease: risk assessment and reduction, including lipid modification [NICE guideline NG238]. https://www.nice.org.uk/guidance/ng238.

[18] University of Oxford University of Oxford research data policy. https://researchdata.ox.ac.uk/university-oxford-data-management-policy.

[19] Braun V, Clarke V (2006). Using thematic analysis in psychology. Qual Res Psychol.

[20] Ziebland S, McPherson A (2006). Making sense of qualitative data analysis: an introduction with illustrations from DIPEx (personal experiences of health and illness). Med Educ.

[21] Watt D (2015). On becoming a qualitative researcher: the value of reflexivity. Qual Rep.

[22] Stacey D, Volk RJ, IPDAS Evidence Update Leads (Hilary Bekker, Karina Dahl Steffensen, Tammy C. Hoffmann, Kirsten McCaffery, Rachel Thompson, Richard Thomson, Lyndal Trevena, Trudy van der Weijden, and Holly Witteman) (2021). The International Patient Decision Aid Standards (IPDAS) Collaboration: evidence update 2.0. Med Decis Making.

[23] Elwyn G, Vermunt N (2020). Goal-based shared decision-making: developing an integrated model. J Patient Exp.

[24] Montori VM, Ruissen MM, Hargraves IG (2023). Shared decision-making as a method of care. BMJ Evid Based Med.

[25] O’Connor AM, Stacey D, Rovner D (2001). Decision aids for people facing health treatment or screening decisions. Cochrane Database Syst Rev.

[26] Joseph-Williams N, Elwyn G, Edwards A (2014). Knowledge is not power for patients: a systematic review and thematic synthesis of patient-reported barriers and facilitators to shared decision making. Patient Educ Couns.

[27] Elwyn G, Price A, Franco JVA, Gulbrandsen P (2023). The limits of shared decision making. BMJ Evid Based Med.

[28] Joseph-Williams N, Abhyankar P, Boland L (2021). What works in implementing patient decision aids in routine clinical settings? A rapid realist review and update from the International Patient Decision Aid Standards Collaboration. Med Decis Making.

[29] Siemieniuk RA, Agoritsas T, Macdonald H (2016). Introduction to BMJ rapid recommendations. BMJ.

[30] Scottish Government Effective Prescribing and Therapeutics Division (2018). Polypharmacy guidance:realistic prescribing Online: Healthcare Improvement Scotland. https://www.gov.scot/publications/polypharmacy-guidance-appropriate-prescribing-making-medicines-safe-effective-sustainable-2026-2029/.

[31] McCartney M, Connolly K, Sullivan F (2024). Where do UK clinicians find information at the point of care? A pragmatic, exploratory study. BMC Prim Care.

[32] MacWalter G, McKay J, Bowie P (2016). Utilisation of internet resources for continuing professional development: a cross-sectional survey of general practitioners in Scotland. BMC Med Educ.

[33] Maggio LA, Aakre CA, Del Fiol G (2019). Impact of clinicians’ use of electronic knowledge resources on clinical and learning outcomes: systematic review and meta-analysis. J Med Internet Res.

[34] Dawes M, Summerskill W, Glasziou P (2005). Sicily statement on evidence-based practice. BMC Med Educ.

[35] Tikkinen KAO, Guyatt GH (2021). Understanding of research results, evidence summaries and their applicability—not critical appraisal—are core skills of medical curriculum. BMJ Evid Based Med.

